# Effect of Encoding on Prospective Memory

**DOI:** 10.3389/fpsyg.2021.701281

**Published:** 2022-01-24

**Authors:** Youzhen Chen, Manman Zhang, Cong Xin, Yunfei Guo, Qin Lin, Zhujun Ma, Jinhui Hu, Weiting Huang, Qianfang Liao

**Affiliations:** ^1^Department of Psychology, Fujian Normal University, Fuzhou, China; ^2^School of Psychology, Nanjing Normal University, Nanjing, China; ^3^Department of Education, Henan University, Kaifeng, China

**Keywords:** prospective memory, implementation intention encoding, specific encoding, non-specific encoding, visual encoding, auditory encoding

## Abstract

Event-based prospective memory (ProM) refers to remembering to execute planned actions in response to a target ProM cues. Encoding modality influences ProM performance; visual encoding has been studied more than auditory encoding. Further, it has not yet been examined whether different encoding may influence ProM performance in different encoding modalities. This study examines the effects of encoding modality (visual vs. auditory), cue-encoding specificity (specific cue vs. non-specific cue), and encoding modes (standard vs. implementation intention) on event-based ProM tasks. In Experiment 1, cue specificity and encoding modality were manipulated as a within-groups encoding of visual cues is more commonly and between-groups variable. Results revealed the facilitative effect of cue specificity on ProM performance. Also, with respect to encoding modality, participants showed better performance when receiving auditory instructions compared with the visual encoding condition. In Experiment 2, as in Experiment 1, cue specificity and encoding modality were manipulated. Encoding mode was added as a new between-group variable. Result revealed that there was a significant interaction between encoding modality and encoding modes. Visual implementation intention encoding was a more effective method for improving ProM performance compared with visual standard encoding. Furthermore, there was a significant interaction between cue-encoding specificity and encoding modes. Implementation intention encoding enhances ProM performance in non-specific cue-encoding conditions. Overall, the present study found that (1) auditory encoding modality showed superior ProM performance compared with visual encoding, although implementation intention had facilitative on ProM performance regardless of the encoding modalities, and (2) there was better ProM performance under specific encoding compared with non-specific encoding, and implementation intention had a facilitative effect on ProM performance in the non-specific condition.

## Introduction

Prospective memory (ProM) is a memory of an action that refers to executing a delayed intended action in the appropriate context or at a planned time (Sanjram and Khan, 2011). ProM, such as sending a letter when you pass a mailbox, is fundamental to our lives. ProM includes four processes, namely, encoding, maintaining, retrieving intentions, and executing an intended action in the future. The previous ProM studies focused primarily on the involvement in maintenance and retrieval processes in performing a delayed intended action. Only recently have researchers started to explore encoding strategies but mostly focused on visual encoding, and the auditory encoding modality process remains somewhat neglected. The present study focuses on the encoding process, as encoding is a necessary precondition for successfully executing a ProM task.

In laboratory studies, ProM manipulation of encoding is conventionally achieved by providing instructions to participants. Participants were informed of intentions through a verbal or written introduction (Cai and Dong, 2012; Li et al., 2016). Regarding information processing, auditory and visual stimuli are the two most common and independent ways through which humans receive information. The visual pathway leads from the occipital cortex to the inferior temporal cortex, and the auditory pathway leads from the superior temporal cortex to the ventral prefrontal regions (Maeder et al., 2001). The separation of process streams may affect ProM encoding differently. Vedhara et al. (2004) randomly allocated participants to one of four habitual ProM task conditions, namely, no cues, visual cues, auditory cues, or dual cues (auditory and visual cues), of which the last three conditions were operated by visual, auditory, or dualhabitual ProM cue introductions reminder. The results showed that habitual ProM of participants was optimal in the dual-cue condition, suggesting this condition was the most beneficial to habitual ProM performance. Additionally, participants performed better under auditory than visual cue conditions. However, Yang et al. (2008) confirmed that there was no difference between auditory and visual encoding on activity-based ProM for undergraduates, regardless of whether ProM intention was important. Both studies on the effect of the ProM encoding modality have produced inconsistent results. It may be caused by the heterogeneity of subjects in the two studies and the different control of the experiment. Vedhara et al. (2004) adopted a natural experiment with old adults with type 2 diabetes as participants; Yang et al. (2008) adopted a laboratory experiment with young adults as participants. Therefore, it is necessary to explore whether auditory encoding is different from visual encoding. Chen et al. (2014) found that the frontal lobe was activated and engaged in monitoring ProM targets. The auditory pathway and ProM task involved the same brain region—the frontal lobe, so we hypothesized that ProM performance would be better under the auditory encoding modal.

People commonly give specific instructions, but they can also give unclear instructions. Both instructions consist of cue-encoding specificity. Previous research showed that participants perform better in ProM tasks under conditions where they could encode visual specific cues (e.g., “jaguar,” “lion,” and “tiger”) rather than visual general cues (e.g., “animal”; Einstein et al., 1995). Kuhlmann and Rummel (2014) confirmed that intentions encoded by visual specific cues form a tight encoding trace, allowing participants to flexibly allocate cognitive resources, thereby enhancing ProM performance (Kuhlmann and Rummel, 2014). For encoding, these previous studies (e.g., Ball and Bugg, 2018) showed a “specific advantage”, indicating that the memory content comprised mostly specific events with a high proportion of specific memories; to recall this content, there was an advantage to remembering specific details (Chen, 2013). Furthermore, Scullin et al. (2018) systematically investigated the encoding process for ProM and confirmed that specific was better than non-specific cue encoding, for which 22.5% of participants gave little thought to the ProM tasks and tended to translate categories to specific exemplars (Pereira et al., 2018). It may be that participants using non-specific cue encoding had to pay closer attention than those using specific cue encoding to correctly determine ProM targets.

However, all of the above studies compared visually specific and non-specific conditions and did not provide direct evidence of auditory specificity encoding. In the current study, we tested the effects of encoding specificity and encoding modality with undergraduate students, building on these different findings. In the first experiment, we investigated whether there was a specific advantage both in the visual and auditory encoding process and whether non-specific auditory encoding was better than non-specific visual encoding in ProM.

We hypothesized that a manipulation, which reduces cognitive resource requirements by enhancing both visual and auditory target cue specificity, would improve ProM, and the performance in the auditory-specific encoding is better than in the visual-specific encoding. Additionally, we hypothesized that participants would perform better in the auditory non-specific content than in the visual non-specific content.

## Experiment 1

### Methods

#### Participants

The sample size was based on an *a priori* power analysis using the GPOWER 3 software. The effect size *f* was based on previous research (Pereira et al., 2018). The alpha level was 0.05, power was 0.95, and an effect size of 0.5 was considered. To find a statistically significant effect in the model, 54 participants would be necessary. Thus, the goal sample size was 60, to account for dropouts.

The initial sample included 60 undergraduate students (*M*_age_ = 20.55 years, range 18–25; 27.12% males). Each participant had normal or correct-to-normal vision and audition, and none had previously taken part in a similar experiment. Four participants were excluded from the analysis because their parameter estimates of the ongoing task or their ProM task were more than three standard deviations (SDs) from the mean of their respective group. Thus, there were 56 participants in total (*N*_auditory_ = 29, *N*_visual_ = 27).

Participants provided signed informed consent before the experiment. The only demographic information collected from the participants was their age and gender; no names or personal information were recorded. Participants were given a small gift as compensation.

#### Experimental Design

The experiment had a two encoding modality (visual vs. auditory) × two cue-encoding specificity (specific cue vs. non-specific cue) mixed factorial design with the second factor as a within-groups variable. Participants received verbal instructions through earphones and written texts for auditory modality and visual modality, respectively. Instructions were provided until participants fully understood them. The within-subjects variable was counter-balanced among participants to avoid a practice effect. Half of the participants started with the specific cue-encoding block, and the other half started with the non-specific cue-encoding block.

#### Materials

The experimental stimuli were 20 capital English letters (excluding “A,” “E,” “I,” “O,” “F,” and “J”). The letters “A,” “E,” “I,” and “O” were excluded to balance-specific and non-specific cue conditions, and “F” and “J” were omitted because they were reaction keys for the ongoing task. The experimental trials were presented visually in a random order. The ProM target was presented six times. To match the number of presentations of specific and non-specific cues, the ProM cues were the same in all specific conditions. Whether the cue was specific or not, we set “U” as the target. Both visual and auditory ProM targets were used for non-salient cues, to avoid the interference effects of cue salience (see Kliegel et al., 2013). All items were presented in white, Courier New, 60-point font on a black background.

#### Experimental Task

Throughout the experimental task, participants performed two tasks simultaneously, namely, the ongoing task and the ProM task. The ongoing task was a one-back task, in which participants needed to compare the present letter with the previous one and then press “J” if they were the same and “F” if they were different. In the ProM task, participants were asked to press the spacebar for a ProM target.

#### Procedure

This experiment was programmed using the E-Prime 1.1 software. All participants were tested individually in a quiet environment. Participants were initially told that the research goal was to study people’s performances on various computer-based tasks and gauge their thoughts during those tasks.

**FIGURE 1:**
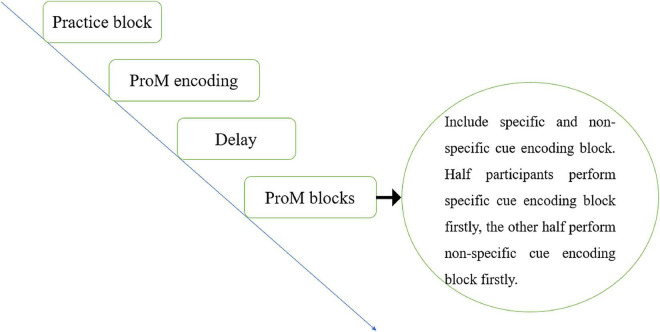
The procedure for Experiment 1.

In the first step, participants performed a practice block (see Figure 1). They received instructions for the one-back task (i.e., ongoing task). For each trial, a fixation cross (+) was presented for 500 ms, followed by a letter for 3,000 ms. The letter would disappear once the participant responded or 3,000 ms had elapsed. Participants received feedback on their accuracy. The “yes” and “no” response keys decisions were counterbalanced, with each decision taking up an equal proportion of the trials.

After participants completed 50 practice trials, they received instructions for simultaneously completing the ProM and ongoing tasks in the normal experimental blocks, i.e., the intention encoding phase. In the visual-specific encoding condition, instructions were presented in the form of text, and encoding content was as follows: “The vowel U may appear during the block. When you see it, you do not need to compare whether the letter is the same as the previous one. Press the spacebar directly.” In the visual non-specific encoding condition, instructions were also presented in the form of text, and encoding content was as follows: “Vowels may appear during the block. When you see a vowel, you do not need to compare whether the letter is the same as the previous one. Press the spacebar directly.” For the audio-specific encoding condition, instructions were played in the form of voice (sound files were embedded in E-prime), and encoding content was the same as the visual-specific encoding condition. When assigned to audio non-specific encoding condition, participants were also played instructions in the form of voice, and encoding content was the same as visual non-specific encoding condition.

Then, we asked participants to repeat the instructions to the experimenter to demonstrate if they understood them. To prevent participants from monitoring the ProM target letters, participants were told that the ProM and ongoing tasks were equally important.

Following the ProM encoding process (i.e., ProM instructions in the different conditions), participants completed an interference task of simple digital arithmetic and then began the formal experiment, which included 94 trials of the ongoing task and six presentations of the ProM target (“U”) for per block. The non-specific and specific cue-encoding blocks were the same, with the exception that the ProM instructions required participants to press the spacebar for vowels. Between the specific and non-specific encoding blocks, there were an interference task and a 2-min rest period.

### Results

For all analyses, the alpha level was set at 0.05. Unless otherwise noted, the dependent variables were reaction time (RT) and accuracy in the ProM target and ongoing trials.

#### Prospective Memory Task Performance

Prospective memory hits occurred when a participant pressed the spacebar on the ProM target trials. ProM performance was defined as the number of hits divided by the number of target events. The means and SDs are presented in Table 1.

**TABLE 1:** The performance of dual tasks in different encoding modalities and encoding specificity *M (SD)*.

Task conditions	ProM tasks		Ongoing tasks	
	ACC	RT (ms)	ACC	RT (ms)
Visual specific	0.78 (0.17)	770 (148)	0.92 (0.06)	670 (138)
Visual non-specific	0.64 (0.35)	828 (215)	0.91 (0.07)	759 (166)
Auditory specific	0.87 (0.16)	820 (155)	0.92 (0.06)	710 (132)
Auditory non-specific	0.75 (0.29)	831 (206)	0.90 (0.08)	740 (201)

*ACC, accuracy; ProM, prospective memory; RT, reaction time.*

To examine the ProM hit, we conducted a two encoding modality (visual vs. auditory) × two (specific vs. non-specific) mixed analysis of variance (ANOVA) with the last variable as a within-groups variable. There was a significant main effect of encoding modality, *F*(1,54) = 5.67, *p* = 0.021 < 0.05, η*_*p*_*^2^ = 0.10; the accuracy in the auditory encoding modality was higher than visual encoding modality. Results also showed a significant main effect of cue-encoding specificity, *F*(1,54) = 6.65, *p* = 0.013 < 0.05, η*_*p*_*^2^ = 0.11. As Table 1 indicates, the accuracy in specific cue-encoding condition was significantly higher than the non-specific cue-encoding condition. There was no significant interaction effect for encoding modality and cue specificity, *F*(1,54) = 0.03, *p* = 0.87.

A 2 × 2 mixed ANOVA was conducted with RTs for correct responses to ProM targets as the dependent variable. There were no significant main effects or interactions, *Fs* < 0.44, *ps* > 0.24.

#### Ongoing Task Performance

The results of both accuracy and RT data are displayed in Table 1. To analyze the performance of the ongoing task, we conducted a two (encoding modality: visual vs. auditory) × two (cue-encoding specificity: specific cue vs. non-specific cue) mixed factorial ANOVA with the last variable as the within-subjects variable and with the ongoing task accuracy and RTs as dependent variables separately.

The analysis of ongoing task accuracy showed that there were no significant main effects of cue encoding modality and cue-encoding specificity, and no interaction effect between cue encoding modality and cue-encoding specificity, and no interaction effect between cue specificity and encoding modality, *F*(1,54) < 2.92, *ps* > 0.09.

The analysis of ongoing task RTs revealed a main effect of cue-encoding specificity, *F*(1,54) = 7.29, *p* = 0.009 < 0.05, η*_*p*_*^2^ = 0.12, suggesting that participants made yes-or-no decisions more faster during the specific cues encoding blocks compared with non-specific cues encoding blocks in both the auditory and visual conditions. There were no other significant differences in RT between the auditory and visual encoding modalities, *F*(1,54) = 0.09, *p* = 0.77 > 0.05, and no interaction effect between cue-encoding specificity and encoding modality, *F*(1,54) = 1.83, *p* = 0.18 > 0.05.

### Discussion

Notably, there was a significant difference in ProM performance of visual and auditory encoding in Experiment 1. Participants had a higher accuracy in the auditory encoding condition than in the visual encoding condition, which was consistent with our hypothesis and confirmed our suspicion that auditory encoding was more convenient. Auditory encoding information is transmitted to the ventral prefrontal regions (Maeder et al., 2001), which happens to be the lobe activated by the ProM intention. The common lobe may make auditory encoding more advantageous. However, the discovery was inconsistent with the conclusion of Yang et al. (2008), who concluded that participants could successfully complete ProM tasks regardless of whether they used visual encoding or auditory encoding, as long as they formed the correct ProM intention. The reason for the different results may be that Yang et al. (2008) explored the effect of encoding on an activity based on ProM, which did not require pausing ongoing tasks and was relatively simple. However, our study explored the effect of encoding on event-based ProM, which required participants to pause ongoing tasks and translate recognitive cost to the ProM task. The difficulty of ProM task may be an important factor.

In Experiment 1, we found advantages of specific cue encoding for ProM, which was consistent with our hypothesis and in line with Einstein et al. (1995) and Hicks et al. (2005), that participants can detect more ProM targets in the specific condition than in non-specific intentions. Participants performed significantly faster in ongoing tasks in the specific condition relative to the non-specific condition, which was consistent with the conclusion of Hicks et al. (2005) that non-specific intentions caused more task interference than specific intentions to the ongoing task. However, RTs of ProM tasks have no significant differences, suggesting possessing two intentions (i.e., ProM intention and ongoing task intention) does not exert the same costs as each would exert individually; the slow RTs of the ongoing task do not necessarily preserve ProM performance (Hicks et al., 2005).

Our finding indicated that participants in the specific cue-encoding condition performed better than those in the non-specific cue-encoding condition, and the RTs of the ongoing task were shorter in the specific cue-encoding condition than in the non-specific cue-encoding condition, which somewhat differs from those of Scullin et al. (2018), who found that some ProM encoding components can be done “in passing” (i.e., a perfunctory and transient manner). We can infer that the encoding process requires cognitive resources, and specific cue encoding is more effective than non-specific cue encoding. Although Scullin et al. (2018) were more focused on thought probes during ProM encoding instead of RTs for retrieval and manipulated only non-specific encoding, their results were consistent with those of the present study in suggesting that specific encoding is more convenient and efficient than non-specific encoding, regardless of cue focality. Since non-specific cue encoding is a common and unavoidable phenomenon, we conducted Experiment 2 to investigate how to improve ProM performance using non-specific cue encoding.

Taken together, the results of Experiment 1 showed that auditory encoding was superior to visual encoding, and specific cue encoding was superior to non-specific cue encoding in terms of ProM intention.

## Experiment 2

In light of the findings of Experiment 1 concerning inferior performance, Experiment 2 further investigated how implementation intention could improve ProM performance under visual and non-specific encoding conditions. Meeks and Marsh (2010) reported that the intention encoding of visual implementation was an efficient encoding method. Thus, we performed Experiment 2 to explore whether implementation intention could improve performance on ProM tasks in the visual and non-specific encoding conditions.

Implementation intention encoding is a conscious formation of a specific intention and a response to a specific stimulus (Gollwitzer and Peter, 1999; Scott, 2016) that determines when, where, and how to put a goal into action based on the goal intention. Implementation intention encoding consisted of a typical statement in the form of “if situation x occurs, then I will perform intended action y.” The statement was often accompanied by asking participants to mentally visualize (usually for 30 s) the intended action and repeating the implementation intention instructions to the researcher. McFarland and Glisky (2012) argued that the verbal articulation of implementation intention is sufficient to improve ProM performance, and imagery instruction is unnecessary. Scullin et al. (2017) reported that a verbal statement and an imagery procedure for implementation intention significantly increased the generation of high typicality exemplars, suggesting that verbally repeating instructions and imagery procedures for implementation intention produced the same results. However, imagining the context of implementation intention is more difficult to control and may result in additional variables (Brewer et al., 2011). Therefore, the present study asks participants to repeat ProM introductions two times (Guo et al., 2016). Conversely, in standard encoding condition (hereafter referred to as standard encoding in this study), participants are told what they should do but without asking them to form an implementation intention or other intention encoding (Mcdaniel et al., 2008; Meeks and Marsh, 2010; Rummel et al., 2012), and the researchers check with participants to make sure they know what to do without an articulated strategy or approach to the task. Implementation intention differs from standard encoding in that it asks participants to consciously encode ProM targets and ProM intended behaviors, which forges connections between ProM targets and actions and does not utilize the cognitive resources of ongoing tasks.

By enhancing the automated connection between ProM cues and actions, implementation intention has been shown to improve ProM performance (Chasteen et al., 2010; Zimmermann and Meier, 2010; Rummel et al., 2012; Chen et al., 2015; Guo et al., 2016). For example, Lin (2016) found that compared with standard encoding, implementation intention improved the accuracy of ProM but not the RTs, regardless of cognitive load. Lv (2010) compared the differences between implementation intention and standard encoding, concluding that implementation intention strengthened the relationship between ProM cues and responses to ProM targeted actions, contributing to participants performing ProM tasks automatically without cognitive resources.

Above all, existing research has focused on intention encoding of visual implementation in the specific cue-encoding condition and justified that the intention of visual implementation was useful for enhancing ProM; however, it remains unclear whether auditory implementation intention encoding is as effective as visual implementation intention encoding and whether the advantage of implementation intention can be generalized to the non-specific cue-encoding condition. Experiment 2 hypothesize that implementation intention can improve ProM performance in the visual and non-specific encoding conditions, and auditory implementation intention is also an efficient encoding method.

### Methods

#### Participants

The sample size was based on an *a priori* power analysis using the GPOWER 3 software. The effect size *f* was based on previous research (Pereira et al., 2018). The alpha level was 0.05, power was 0.95, and a size effect of 0.5 was considered. To find a statistically significant effect in the model, 76 participants would be necessary. A goal sample size of 120 was set to account for dropouts.

Experiment 2 was conducted with 120 undergraduate students (*M*_age_ = 20.64, range: 18–26; 32.20% males) who received a gift for participating. Two participants were excluded because their RT data were ± 3 SDs from mean of their group. There were 62 participants in the implementation intention conditions (i.e., 30 in the visual condition and 32 in the audio condition) and 56 participants in the standard encoding conditions (i.e., 27 in the visual condition and 29 in the audio condition). The other conditions were the same as Experiment 1.

#### Design

The experiment had a two encoding modality (visual vs. auditory) × two encoding modes (standard vs. implementation intention) × two cue-encoding specificity (specific cue vs. non-specific cue) mixed factorial design with the last factor as the within-groups variable. The cue specificity order was balanced among participants: half executed the specific cue condition first, while the other half executed the non-specific cue condition first. RTs and accuracy rates served as the dependent measures.

#### Materials

We used an Acer computer and a 14.5-inch CRT display. The experiment was programmed using the E-Prime 1.1 software. All details regarding the stimuli were consistent with Experiment 1.

#### Procedure

The instructions given to participants describing the one-back task were almost identical to those in Experiment 1, with the only difference that participants were encoded by implementation intention in the implementation intention condition. For both the auditory and visual implementation intention conditions, participants were asked to repeat the implementation intention instructions two times. The implementation intention instructions were, “If you see any of the vowels, press the spacebar directly” or “If you see the letter U, press the spacebar directly.” The standard condition instructions were, “The letter U may appear during the experiment. When you encounter the letter U, it is not necessary to compare whether the letters are the same or not, just press the spacebar.” Participants in the standard encoding condition were not required to repeat the instructions about the ProM task. The remaining instructions were the same as in Experiment 1.

### Results

#### Prospective Memory Task Performance

Table 2 shows the means and SDs for ProM tasks. An ANOVA was carried out for (encoding modality: visual vs. auditory) × two encoding modes (standard vs. implementation intention) × two (cue-encoding specificity: specific cue vs. non-specific cue) mixed factorial design with the last factor as within-groups variable on ProM accuracy. Results yielded a significant main effect of encoding specificity, *F*(1,114) = 5.02, *p* = 0.027 < 0.05, η*_*p*_*^2^ = 0.04, indicating that the accuracy rate was significantly higher in the specific cue-encoding condition (*M* = 0.82, *SD* = 0.02) than in the non-specific cue-encoding condition (*M* = 0.75, *SD* = 0.30).

**TABLE 2:** The performance of ProM tasks in different conditions *M (SD)*.

Encoding models	Encoding modality	Encoding specificity			
		Specific encoding		Non-specific encoding	
		ACC	RT (ms)	ACC	RT (ms)
Standard encoding	Audio	0.87 (0.16)	820 (155)	0.75 (0.29)	831 (207)
	Visual	0.78 (0.17)	771 (148)	0.64 (0.35)	828 (216)
Implementation intention	Audio	0.78 (0.23)	756 (166)	0.77 (0.29)	832 (192)
	Visual	0.84 (0.17)	780 (131)	0.83 (0.22)	913 (186)

We found a marginal significant interaction between encoding mode and encoding specificity, *F*(1,114) = 3.40, *p* = 0.068, η*_*p*_*^2^ = 0.03. Further simple effects analyses revealed that there was a significant difference between implementation intention and standard encoding in the non-specific cue-encoding condition, *F*(1,114) = 4.08, *p* = 0.046 < 0.05, η*_*p*_*^2^ = 0.04, suggesting that implementation intention (*M* = 0.80, *SD* = 0.04) had a higher accuracy than standard encoding (*M* = 0.81, *SD* = 0.02). There was no significant difference between implementation intention and standard encoding in the specific cue-encoding condition, *p* = 0.69 > 0.05. In the standard encoding condition, there was a significant difference between the specific cue-encoding and non-specific cue-encoding condition, *F*(1,114) = 7.94, *p* = 0.006 < 0.05, η*_*p*_*^2^ = 0.07, suggesting that specific cue-encoding condition (*M* = 0.83, *SD* = 0.03) had higher accuracy than non-specific cue encoding (*M* = 0.69, *SD* = 0.04). There was no significant difference between the specific cue encoding and non-specific cue encoding condition in the implementation intention encoding condition, *p* = 0.78 > 0.05.

We also found a significant interaction between encoding mode and encoding modality, *F*(1,114) = 7.56, *p* = 0.007 < 0.05, η*_*p*_*^2^ = 0.07. The further simple effects analysis found there was a significant difference between standard encoding and implementation intention encoding in the visual encoding condition, *F*(1,114) = 8.85, *p* = 0.004 < 0.05, η*_*p*_*^2^ = 0.07, suggesting that participants had a higher ProM accuracy in the implementation intention encoding condition (*M* = 0.81, *SD* = 0.03) than the standard encoding condition (*M* = 0.71, *SD* = 0.03). In the auditory encoding condition, there was no significant difference between standard encoding and implementation intention encoding, *p* = 0.38 > 0.05. We found a significant difference between the visual and auditory encoding condition in the standard encoding condition, *F*(1,114) = 5.38, *p* = 0.02 < 0.05, η*_*p*_*^2^ = 0.05, suggesting that participants had higher ProM accuracy in the auditory encoding condition (*M* = 0.81, *SD* = 0.03) than the visual encoding condition (*M* = 0.71, *SD* = 0.03). In the implementation intention encoding condition, there was no significant difference between the visual and auditory encoding condition, *p* = 0.12 > 0.05. There were no other significant main effects or interaction effects, *Fs* < 2.34, *ps* > 0.13.

Prospective memory RT of correct responses (i.e., trials where participants correctly responded to the PM targets) were analyzed by performing an ANOVA for two encoding modality (visual vs. auditory) × two encoding modes (standard vs. implementation intention) × two cue-encoding specificity (specific cue vs. non-specific cue) mixed factorial design with last factor as within-groups variable. There was a significant main effect of cue specificity, *F*(1,114) = 11.88, *p* = 0.001 < 0.05, η*_*p*_*^2^ = 0.09, indicating RTs were much faster in the specific cue condition (*M* = 787, *SD* = 13.92) versus the non-specific cue condition (*M* = 851, *SD* = 18.43). There were no other significant main effects or interaction effects, *Fs* < 2.77, *ps* > 0.09.

#### Ongoing Task Performance

To analyze performance in the ongoing task, we conducted a two encoding modality (visual vs. auditory) × two encoding modes (standard vs. implementation intention) × two cue-encoding specificity (specific cue vs. non-specific cue) mixed factorial design with the last factor as within-groups variable on accuracy and RT of ongoing task, respectively. Table 3 shows the means and SDs of ongoing task trials.

**TABLE 3:** The performance of ongoing tasks in different conditions *M (SD)*.

Encoding models	Encoding modality	Encoding specificity			
		Specific encoding		Non-specific encoding	
		ACC	RT (ms)	ACC	RT (ms)
Standard encoding	Audio	0.92 (0.06)	710 (132)	0.90 (0.08)	740 (201)
	Visual	0.92 (0.06)	670 (139)	0.91 (0.07)	759 (166)
Implementation intention	Audio	0.92 (0.05)	671 (142)	0.90 (0.06)	732 (169)
	Visual	0.94 (0.04)	703 (138)	0.92 (0.05)	805 (162)

The analysis of accuracy of ongoing tasks found that the only significant finding was a main effect of cue-encoding specificity, *F*(1,114) = 10.33, *p* = 0.002 < 0.05, η*_*p*_*^2^ = 0.08, showing a higher accuracy on specific cues (*M* = 0.92, *SD* = 0.01) compared with non-specific cues (*M* = 0.91, *SD* = 0.01). There were no other significant main or interaction effects, *Fs* < 1.85, *ps* > 0.17.

Results of RT of ongoing tasks yielded a significant main effect of cue specificity, *F*(1,114) = 21.32, *p* = 0.000 < 0.001, η*_*p*_*^2^ = 0.16, with participants demonstrating faster RTs in the specific (*M* = 689, *SD* = 12.70) than the non-specific condition (*M* = 759, *SD* = 16.15). There were no other significant main or interaction effects, *Fs* < 2.67, *ps* > 0.11.

### Discussion

Consistent with previous studies (e.g., Scullin et al., 2017) and our hypothesis, Experiment 2 confirmed that visual implementation intention was effective for improving ProM. The result of Experiment 2 showed there was a significant difference in ProM accuracy between visual implementation intention encoding and visual standard encoding, indicating that visual implementation intention can improve ProM accuracy relative to visual standard encoding.

We did not find specificity advantage in the implementation intention encoding condition, but we found participants had a higher ProM accuracy in the implementation intention encoding condition than the standard encoding condition in the non-specific cue-encoding condition, which was consistent with our hypothesis and suggested that implementation intention can improve the performance in the non-specific encoding condition and extent the finding that implementation intention was suitable to non-specific cue encoding. We found no significant difference between the standard encoding and implementation intention encoding condition in specific cue encoding, which was consistent with Meeks and Marsh (2010), who found that specific cues (e.g., “deer” and “cow”) led to a ceiling effect in both implementation intention (imagery; imagery + when-then) and conventional event-based specific cue ProM conditions; thus, there was no opportunity to investigate benefits of implementation intention. Both Meeks and Marsh (2010) and the present study demonstrated that non-specific implementation intention can improve ProM performance.

The current finding of no significant difference of ongoing tasks in different encoding modes was somewhat in line with Lv (2010), who found that under implementation intention conditions, reduced ongoing task performance did not improve ProM task performance. The finding that implementation intention did not reduce ongoing task performance of participants relative to standard instructions contrasted Chen et al. (2015), who reported that individuals had longer RTs for ongoing tasks when encoded by implementation intention. We speculated that this disparity could be explained by the relatively simple ongoing task used in the current study; the further study can manipulate the two-back task as the ongoing task.

## General Discussion

The result of Experiment 2 showed that there was a significant difference between the visual and auditory modalities in the standard encoding condition, which was consistent with Experiment 1 that proved that auditory standard encoding was more useful than visual standard encoding. The finding confirmed our hypothesis that auditory encoding and ProM process mechanism share the common lobe, which is beneficial for auditory encoding. However, our results are inconsistent with those of Yang et al. (2008), who demonstrated that encoding modality had no effect on activity-based ProM. Yang et al. (2008) explored whether sensory modality (auditory and visual) influences ProM encoding, by providing different sensory encoding modalities. The results showed that irrespective of whether audio or visual information was encoded, intentions of participants relative to the ProM task were the same. When formed correct intention, participants were able to successfully complete the task in the appropriate amount of time. The current research denied the conclusion and proved the benefit of auditory encoding.

The findings in the two experiments produced a consistent pattern, showing the advantages of specific cues encoding in the standard encoding condition. We purposely used a vowel (“U”) as a ProM target, whether it was used in a specific encoding condition or non-specific encoding condition. This decision was based on situations one might face in everyday life, such as going to the supermarket to buy food for lunch and wondering which purchase would be better. For example, an individual is more likely to purchase fruit when given the name of a specific fruit than when only told to purchase fruit. Our results are consistent with Lourenço et al. (2013), who demonstrated that target specification can reduce costs in non-focal ProM and trial-by-trial changes in task interference, as the result of top-down attention control processes. Previous research suggested that non-specific cue encoding may activate more connections than specific cue encoding; therefore, participants need more cognitive to perform intended tasks and resulting in slower RTs (Hicks et al., 2005; Loft et al., 2008). If target cues are specific and unambiguous, then the connection between target and behavior becomes more specific, thereby facilitating ProM retrieval. The current findings were consistent with Pan (2010), who found that when ProM encoding was specific, participants adopted automatic processing, the attention required relatively few resources, and ProM performance was high. However, when ProM encoding was non-specific, the monitoring processing method was used more often, resulting in a relatively higher resource consumption, affecting ProM performance, and significantly impacting RT and accuracy in the ongoing task.

Compared with non-specific encoding, specific encoding requires fewer cognitive resources. This finding helps to explain why it can be more difficult to perform an intended action without a specific task description. For example, it may be difficult to choose flowers when only told to get “fresh flowers” and not told to select a specific type of flower. There were also significant effects of accuracy and RT for the ongoing task. Participants who were assigned to the specific ProM target condition had more cognitive resources with which to perform the ongoing task than those in the non-specific ProM target condition.

Implementation intention encoding can not only make up for the disadvantages of visual encoding but also improve ProM performance for non-specific encoding conditions. The present study confirmed that implementation intention enhanced goal attainment by facilitating the initiation of planned responses upon encountering critical situations (Gollwitzer and Brandstätter, 1997). The mental representation of the ProM tasks specified in the if-part becomes a highly activated and easily accessible cue. By using implementation intention encoding, individuals establish a vivid psychological image between ProM intention and behavior, which has a superposition effect with words encoded by visual, resulting in that visual implementation intention encoding can significantly improve ProM performance. As a consequence, the ProM task receives attentional and perceptual priority (Achtziger et al., 2011; Janczyk et al., 2015) and is easily detected in the environment. A strong link is forged between ProM cue and ProM response specified in then-part. This renders ProM response automatically (Gan et al., 2017, 2020), and no other cognitive resources are needed to perform theProM task correctly. This study shows that the advantages of implementation intention encoding are not affected by audiovisual encoding modality; moreover, implementation intention encoding can compensate for the shortcomings of visual encoding and promote the execution of ProM tasks, without the cost of ongoing tasks.

Implementation intention creates a strong mental representation of the situation and a strong linkage between situation and response that makes it easy to execute the behaviors. It helps people to overcome the gaps between the intentions and the actual behaviors, and improves ProM performance (Chen et al., 2015). The empirical data of the present study confirmed that the effect of implementation intention can be generalized to non-specific situations (Bieleke et al., 2018; Huang et al., 2020). Through the connection of implementation intention, the ProM performance under non-specific conditions has been significantly improved, reaching a level equivalent to that under specific conditions. Combined with the fact that there was no significant difference between the implementation intention and standard encoding condition, we inferred that implementation intention can specify intention, which is similar to specify encoding.

### The Limitations of the Present Study

One potential limitation of the present study is that the one-back task was our ongoing task, and almost all participants performed it perfectly, with accuracy rates above 0.90, which demonstrated high ceiling effects. Maybe the one-back task was too easy for undergraduates. Thus, future studies should use more difficult tasks and manipulate task load. Also, we manipulated encoding and neglected the consistency of encoding and extraction modality. It is also worth further research to determine whether there is an audio-visual modality effect on ProM performance, by comparing the consistency of encoding and extraction modality of visual and auditory encoding.

## Conclusion

The results of our study suggest there are specific advantages in the field of ProM, and one such advantage is that specific encoding can contribute to ProM performance relative to non-specific encoding. Also, implementation intention is an effective method for enhancing ProM performance in a visual encoding condition and in a non-specific encoding condition. Regardless of implementation intention or standard encoding, individuals could successfully complete tasks as long as they were encoded by specific intention.

## Data Availability Statement

The original contributions presented in the study are included in the article/supplementary material, further inquiries can be directed to the corresponding author.

## Ethics Statement

The studies involving human participants were reviewed and approved by the Ethics Committee of Fujian Normal University. The patients/participants provided their written informed consent to participate in this study.

## Author Contributions

MZ performed the analysis and was responsible for the design and planning of the study. YC, MZ, CX, YG, QL, ZM, JH, WH, and QFL performed the study. MZ and YC wrote the final report. All authors contributed to the article and approved the submitted version.

## Conflict of Interest

The authors declare that the research was conducted in the absence of any commercial or financial relationships that could be construed as a potential conflict of interest.

## Publisher’s Note

All claims expressed in this article are solely those of the authors and do not necessarily represent those of their affiliated organizations, or those of the publisher, the editors and the reviewers. Any product that may be evaluated in this article, or claim that may be made by its manufacturer, is not guaranteed or endorsed by the publisher.
